# Marginal Zone B-Cell Populations and Their Regulatory Potential in the Context of HIV and Other Chronic Inflammatory Conditions

**DOI:** 10.3390/ijms23063372

**Published:** 2022-03-21

**Authors:** Kim Doyon-Laliberté, Matheus Aranguren, Johanne Poudrier, Michel Roger

**Affiliations:** 1 Centre de Recherche du Centre, Hospitalier de l’Université de Montréal (CRCHUM), Tour Viger 900 Rue St-Denis, Montréal, QC H2X 0A9, Canada; kim.doyon-laliberte@umontreal.ca (K.D.-L.); mat.aranguren@gmail.com (M.A.); 2 Département de Microbiologie, Infectiologie et Immunologie de l’Université de Montréal, Montréal, QC H2X 0A9, Canada

**Keywords:** Bregs, marginal zone (MZ) B-cells, B-cell activating factor (BAFF), HIV

## Abstract

Inflammation in the context of Human Immunodeficiency Virus (HIV) establishes early and persists beyond antiretroviral therapy (ART). As such, we have shown excess B-cell activating factor (BAFF) in the blood of HIV-infected progressors, as soon as in the acute phase, and despite successful ART. Excess BAFF was associated with deregulation of the B-cell compartment; notably, with increased frequencies of a population sharing features of both transitional immature (TI) and marginal zone (MZ) B-cells, we termed Marginal Zone precursor-like (MZp). We have reported similar observations with HIV-transgenic mice, Simian Immunodeficiency Virus (SIV)-infected macaques, and more recently, with HIV-infected Beninese commercial sex workers, which suggests that excess BAFF and increased frequencies of MZp B-cells are reliable markers of inflammation in the context of HIV. Importantly, we have recently shown that in healthy individuals, MZps present an important regulatory B-cell (Breg) profile and function. Herein, we wish to review our current knowledge on MZ B-cell populations, especially their Breg status, and that of other B-cell populations sharing similar features. BAFF and its analog A Proliferation-Inducing Ligand (APRIL) are important in shaping the MZ B-cell pool; moreover, the impact that excess BAFF—encountered in the context of HIV and several chronic inflammatory conditions—may exert on MZ B-cell populations, Breg and antibody producing capacities is a threat to the self-integrity of their antibody responses and immune surveillance functions. As such, deregulations of MZ B-cell populations contribute to autoimmune manifestations and the development of MZ lymphomas (MZLs) in the context of HIV and other inflammatory diseases. Therefore, further comprehending the mechanisms regulating MZ B-cell populations and their functions could be beneficial to innovative therapeutic avenues that could be deployed to restore MZ B-cell immune competence in the context of chronic inflammation involving excess BAFF.

## 1. Introduction

Marginal zone (MZ) B-cells are innate-like, and possess a polyreactive B-cell receptor (BCR) and several pattern recognition receptors (PRR) [1,2]. They are known to generate low-affinity first-line antibody responses against invading pathogens such as encapsulated bacteria [3]. Important to this Special Issue is the fact that we and others have shown that MZ B-cell populations also possess strong regulatory B-cell (Breg) potential [4]. Unfortunately, deregulations affecting MZ B-cell populations have been reported in the context of Human Immunodeficiency Virus (HIV) and other chronic inflammatory conditions [2,5,6]. In this review, we will only briefly discuss MZ B-cell ontogeny and antibody responses, as these topics have been reviewed elsewhere and are beyond the scope of this work [1,2,3,7]. We will concentrate our efforts on examining the regulatory capacities of MZ and other B-cell populations sharing similar features. The importance of the B-cell activating factor (BAFF) and its analog A Proliferation-Inducing Ligand (APRIL) in shaping the MZ B-cell pool and Breg profile will be discussed. The deregulation of MZ B-cell populations and development of MZ lymphomas (MZL) in the context of HIV and other inflammatory diseases will also be addressed. Lastly, we will talk about possible therapeutic avenues that could be deployed to restore MZ B-cell immune competence.

## 2. Ontogeny of MZ B-Cells

**Figure 1:**
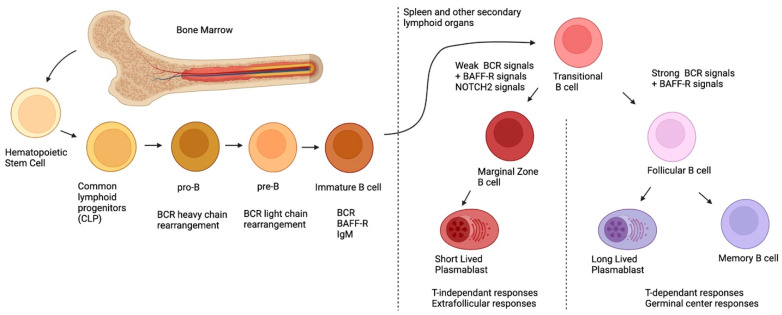
Ontogeny of postnatal B-cells.

The first B-cell progenitors can be found as early as 7 weeks post conception in the fetal liver [8]. However, the B lymphoid progenitor compartment differs between fetal and postnatal life, and herein, we will only briefly focus on postnatal ontogeny, as these topics are beyond the scope of this article and have been thoroughly reviewed elsewhere [8]. As depicted in Figure 1, post-natal B-cell development originates in the bone marrow, first, with pluripotent hematopoietic stem cells and their differentiation into common lymphoid progenitors (CLP) [9]. During their development, B-cells will undergo rearrangement of their BCR heavy chains (during the pro-B stage) and their light chains (during the pre-B stage) via the action of the Recombination-activating genes 1 and 2 (*RAG1*, *RAG2*) [10]. Overall, during these recombination steps, positive and negative selection ensures that the new BCR is functional, yet not autoreactive [11]. After these selection processes, immature B-cells, which express the newly rearranged BCR of the IgM isotype on their surface, exit the bone marrow and migrate to the spleen or other secondary lymphoid organs, where they complete their maturation and differentiation [11]. At this point, the immature B-cell is called transitional immature (TI); it will follow maturation steps comprising stages TI-1 to TI-2 and TI-3, and will commit to either the follicular (FO) or the MZ B-cell fates, depending on the signals it receives (see below) [12,13,14]. Although this generalized sequence of events appears to be similar for the murine and human systems, several distinctions prevail [15]. One major difference is the fact that in the murine system, MZ B-cells are believed to arise mainly from TI-2 progenitors, which complete their maturation in the spleen, where they appear to be restricted in contrast to those observed in the human system where MZ B-cells recirculate [16]. Interestingly, recent studies in humans have demonstrated the existence of bone-marrow-derived TI-2 IgM^lo^ and IgM^hi^ progenitors, the latter of which share transcriptional features with MZ B-cells, express α4β7 and migrate to the gut-associated lymphoid tissue (GALT) [14]. This suggests that the GALT may be an important site for human MZ B-cell differentiation [15].

FO and MZ B-cells are known as conventional B-cells or B2 cells [17]. Another subpopulation with innate-like properties has been identified in mice, and its cells are dubbed B1 cells. B1 and B2 cells differ in their ontogeny, as B1 cells come from a distinct lineage in the fetal liver and B2 cells originate from the bone marrow; moreover, B1 and B2 cells differ in their location, as B1 cells are most commonly found in the pleural cavity, unlike B2 cells [17,18]. Despite the fact that B1 cells are acknowledged in mice, their presence in humans, to date, remains controversial [18].

To date, at least three signals are involved in the FO versus MZ B-cell fate: *1.* via the receptor for BAFF (BAFF-R), fundamental for sending survival signals to TI B-cells and for activating the canonical nuclear factor kappa B (NF-κB) signaling path; *2*. signals resulting from the engagement of the newly expressed BCR; and *3*. Notch Receptor 2 (NOTCH 2) signals, the latter two being responsible for cell fate commitment [13]. When NOTCH2 binds to its ligand, delta-like 1 (DLL1) (expressed by a variety of cells in the spleen such as endothelial cells of the red pulp venule), in the context of weak BCR signaling, the former is internalized and translocated into the nucleus; there, it binds to DNA and allows the expression of genes involved in MZ differentiation [19]. However, strong BCR signaling will induce Bruton’s tyrosine kinase (BTK) signals, which will inhibit the NOTCH2 signaling pathway, allowing for the expression of genes involved in FO differentiation [20]. It is important to note that the level of BCR signaling required for MZ differentiation induces the expression of a disintegrin and metalloproteinase-containing protein 10 (ADAM10); this is required for the cleavage of NOTCH2, necessary for its nuclear translocation [21], implying that a complete absence of BCR stimulation will impede MZ B-cell differentiation. While BAFF itself is not a direct player in MZ differentiation, it can skew the TI B-cells into differentiating into MZ by upregulating NOTCH2 expression [13,22,23].

To recapitulate, MZ B-cells originate from the bone marrow, where they will undergo BCR rearrangement. After expressing the newly arranged BCR and BAFF-R, they will migrate to the secondary lymphoid organs where they will complete their differentiation based on three signals: BAFF-R, BCR and NOTCH2. Weaker BCR signals coupled with NOTCH2 signals will dictate the differentiation towards an MZ profile, whereas strong BCR signals and a lack of NOTCH2 signaling will dictate the differentiation towards an FO profile.

In humans, MZ B-cells are usually found in the marginal zone, a strategic region surrounding germinal centers (GC). As such, MZ B-cells have been observed in the spleen and other secondary lymphoid organs such as tonsils, lymph nodes and the GALT, in areas such as in the sub-endothelial dome of the Peyer’s patches [16,24]. Interestingly, as mentioned above, human MZs have the capacity to recirculate in blood, a trait that has not been identified in their murine counterparts. As such, in mice, MZ B-cells appear to be restricted to the splenic marginal zone, which is at the interface between the red and white pulps, and surrounding the follicular area of the spleen [25]. This difference contributes to fueling the controversy about the MZ’s existence in humans, given that most studies on MZ B-cell biology were conducted in mice and restricted to the spleen. The spleen is one of the most irrigated organs, at any given time receiving around 5 to 10% of the total blood volume, which is huge considering its size and oxygen consumption under steady-state conditions [26]. One of the reasons for this lies in the fact that the spleen is involved in the “screening” of the circulatory system for bloodborne antigens [1]. Indeed, the marginal zone is placed strategically next to the blood entries in the spleen, allowing for MZ B-cells and other innate cells such as neutrophils, dendritic cells (DCs) and macrophages to act as first-line defenders, quickly responding to antigens found in the circulation [1,2].

The B-cell composition of the marginal zone area is heterogeneous, as populations such as B1, memory and MZ B-cells transit to, or reside within, that zone; this renders the characterization of such populations difficult, as they often share several markers. In humans, MZ B-cells are characterized by their high expression levels of the atypical major histocompatibility complex (MHC) class I molecule CD1c; the surface immunoglobulin (Ig)M and the complement receptor CD21; and the low and transient expression of CD23, a C-type lectin which is also the Fc receptor for IgE and is highly expressed by FO B-cells [1]. Interestingly, human MZ B-cells express the memory B-cell marker CD27, and their Igs present signs of somatic hypermutations (SHMs), even though these B-cells mostly produce extra-follicular T-independent Ig responses and, therefore, are not generated from typical T-dependent GC reactions, where Ig SHM and affinity maturation usually take place (discussed below) [27]. As such, MZ B-cells are often referred to as “antigen-experienced” cells [3,25,28]. However, there is some evidence of “memory-like” MZ B-cells that possess a more specific affinity for some bacterial antigens [27]. The fact that MZ B-cells express CD27, together with certain differences between human and mice, makes the classification of MZ B-cells in humans controversial, where some authors consider these cells to be unswitched IgM memory B-cells [3,25,29]; although, several key differences between unswitched IgM memory and MZ B-cells have been documented [25]. The current tendency to track human B-cell populations—especially in blood in the context of inflammation, based on CD27 and CD21 expression levels—makes it difficult to identify innate-like populations such as MZs, as they are of low frequencies and fall into larger groups characterized in bulk. This is likely to preclude any contribution from such rarer populations. To this end, our experience is that the usage of several markers should be more widely applied in order to identify such B-cell populations, whose contribution to inflammation is non negligible, as discussed further below.

## 3. MZ B-Cells and Their Antibody Responses

As first-line defenders, MZ B-cells possess several PRRs such as Toll-like receptors (TLRs) and C-type lectins. Given the polyreactive nature of their BCR, MZ B-cells bear a strong autoreactive potential [7,30,31]. They are known for their quick response against bloodborne pathogens, notably towards encapsulated bacteria [32]. Following their activation, mostly in a T-independent manner (discussed below), they differentiate into short-lived plasma cells that will mostly produce antibodies of the IgM isotype, providing a first level of defense while awaiting a more refined adaptive response from FO B-cells [33,34,35]. To this end, MZ B-cells have the potential to capture bloodborne antigens and then migrate from the marginal zone to the follicles (in a process known as shuttling); from there, they deliver immune-complexed antigens via antibodies through Fc receptors such as CD32, or via the complement system through complement receptors such as CD21 and CD35, to follicular dendritic cells (FDCs) [36]. This process has been found to be fundamental to the generation of GCs.

Briefly, GC reactions are sites of antigen-specific T-dependent—notably via CD40-CD40L signaling—FO B-cell differentiation and Ig affinity maturation [37]. Overall, there are two detectable phases in GC reactions: the dark phase where B-cells, having received signals for class switch recombination (CSR), stop expressing surface Ig and change their isotype into either of IgG, IgE or IgA, in order to gain effector functions (though CSR is not restricted to GC reactions) [38,39,40]. During this stage, B-cells (or centroblasts) proliferate and undertake SHM to increase antibody affinity for the antigen. Following the dark phase, the light phase allows for B-cells, or centrocytes, to express somatically mutated class-switched Ig on their surface with a view to being selected [40]. Notably, this differentiation scheme is not restricted to one round. The selection process is based on Ig affinity for the antigen presented at the surface of FDC, and on signals received by follicular helper T-cells (T_fh_) [40]. B-cells with poor affinity will undergo apoptosis by neglect. This GC process is essential to assure the maturation and selection of memory B-cells and long-lived plasma cells, which guarantee the generation of high affinity antibodies endowed with refined effector potential [36].

As mentioned earlier, while MZ B-cells do not generate such high-affinity antibody responses, Ig produced by these cells have been shown (in humans) to bear low levels of SHM [25,41,42,43]. MZ B-cells also have the potential to undergo CSR from IgM to IgG or IgA following the binding of BAFF to the receptor transmembrane activator and calcium modulator and cytophilin ligand interactor (TACI), which is highly expressed at the surface of MZ B-cells [44,45]. However, these antibodies are considered of low-affinity and of a polyreactive nature, in contrast to those produced through GC reactions. Nevertheless, antibodies produced by first-line populations such as MZ B-cells may be relevant in circumstances of microbial control and mucosal homeostasis, as will be discussed.

Interestingly, MZ B-cells have also been shown to migrate to T-cell zones of secondary lymphoid organs and activate CD4^+^ T-cells [46]. Additionally, MZ B-cells are able to present antigens to invariant natural killer T-cells (iNKT), a type of NKT-cell with a restricted TCR repertoire that can recognize lipidic molecules in the context of atypical MHC class I-like molecules of the CD1 family, widely expressed by MZ B-cells [47,48]. These MZ: iNKT cellular interactions confer activation, notably via the CD40-CD40L pathway [48].

## 4. MZ B-Cell Populations and Their Regulatory Potential

Bregs are involved in the maintenance of tolerance and homeostasis of the immune system. Bregs were originally defined as IL-10 producing B-cells (or B10) in mice [49]. Many groups have since identified different murine Breg subsets that possess anti-inflammatory suppressive mechanisms, mostly mediated by IL-10, such as T2-MZP-B-cells, MZ B-cells and B1a B-cells, amongst others (see Table 1). As in mice, human Breg subsets have been mostly identified based on their IL-10 production capacities [49]. Although IL-10 is an important regulatory cytokine, its production alone is not sufficient to qualify a B-cell population as Breg, since several human B-cell populations are capable of IL-10 production in the context of inflammation and upon stimulation [6,50]. However, in these populations, IL-10 production does not persist in time; therefore, these populations could be falsely identified as true Bregs. Unlike the expression of *Forkhead Box P3* (FoxP3), which is a shared feature of regulatory T-cell populations, there is no single marker reported to identify Breg populations to date [51]. As such, several immunoregulatory markers—such as IL-10, Programmed Death Ligand 1 (PD-L1), CD39 or CD73—have been associated with Breg potential and can help identify true Breg populations (see Table 1). Notably, the overlap of several markers used by different groups could imply that different Breg populations might in fact be more similar than expected.

As mentioned earlier, we have previously characterized a B-cell population sharing characteristics of both MZ and TI B-cells, which we termed “precursor-like MZ B-cells” (MZp) and which bears a CD19^+^IgM^high^CD27^+^CD1c^+^CD21^low^CD10^+^ phenotype [5,6,79]. Our recent work has shown that MZps possess strong regulatory potential due to the Breg molecules that they express (see Table 2). Indeed, besides their strong ex vivo IL-10 expression profile, MZps highly express the nuclear receptors (NR)4A1, NR4A2 and NR4A3, as well as the immunoregulatory molecule CD83 (see below) [4,6]. Furthermore, MZps also express the ectonucleotidases CD39 and CD73, as well as several molecules associated with Breg functions, such as Transform Growth Factor Beta (TGF-β), IL-35, TLR10, Human Leukocyte Antigen G (HLA-G) and PD-L1 [4]. Strikingly, we have found that the Breg function of MZp was directly linked with signals involving CD83, and more recently with the PD-1/PD-L1 signaling path, as discussed below [4].

### 4.1. Importance of NR4As

The NR4As are a family of orphan nuclear receptors, meaning that their endogenous ligand is unknown. There are three known transcription factors in this family: NR4A1 (or Nur77), NR4A2 (or Nurr1) and NR4A3 (or NOR-1), all of which possess a certain degree of homology and redundant functions [80]. Normally, a nuclear receptor must bind to its ligand in order to undergo a conformational change that allows for their DNA binding and subsequent gene transcription. However, it has been shown that the transcription factors of the NR4A family may not need such a ligand, since their natural conformation is constitutively active [80].

Members of the NR4A family are known for their regulatory, anti-inflammatory and pro-apoptotic actions. As a matter of fact, expression of all three known members of the NR4A family is essential for the maintenance of FoxP3 expression by Tregs; moreover, their deficiency converts Treg precursors into autoreactive T-cells, possibly due to the nature of Treg selection [81,82]. The expression of NR4As is quickly upregulated (they are “early induced genes”) following several stimulatory engagements (such as after BCR and TCR stimulation, and even TLR signaling) to control unhindered immune responses, notably in the absence of co-stimulation. Moreover, they participate in the contraction of the immunological response by inducing clonally expanded lymphocytes to undergo apoptosis [83,84]. NR4As have been shown to be upregulated in exhausted T-cells in the context of cancer and chronic infection in mice, suggesting a contribution to the control of immune responses in the context of prolonged and/or excessive immune activation [85,86]. The NR4As are also important for monocyte differentiation, since NR4A1 is essential for the differentiation of intermediate and non-classical monocyte subsets, and monocyte derived dendritic cells (MoDC) are absent in NR4A3 knock-out (KO) mice [87,88,89,90]. Lastly, the NR4As are involved in the expression of immune checkpoint molecules such as PD-L1, further illustrating the immune regulatory function of these molecules [91]. Importantly, NR4As are a part of the cyclic AMP (cAMP) response elements (CREs), the expression of which is modulated by the cAMP binding protein (CREB) [92]. The CREB is involved in the expression of several immunoregulatory proteins and anti-inflammatory molecules such as IL-10, and it is activated by the accumulation of cAMP in the cytosol [93].

One of the molecules whose expression is directly controlled by the NR4A family is the immunoregulatory protein CD83 [94]. Accordingly, we have shown that MZp express high levels of CD83 ex vivo, and their Breg function is related to this molecule, as the administration of a CD83 blocking antibody impedes MZp control of CD4^+^ T-cell proliferation in vitro [4]. CD83 is a protein of the immunoglobulin-like superfamily, whose ligand is unknown. It can be found in a membrane-bound or a soluble manner, both of which seem to play different roles in immunity, with soluble CD83 (sCD83) being involved in immunoregulatory roles [95,96,97]. It has been suggested that, similarly to other B7 family members, it can interact with other CD83 molecules in a homotypic manner, a feat that was demonstrated in DCs [98,99,100]. CD83’s expression and role has been shown in a wide variety of regulatory cell populations. Indeed, it has been shown that sCD83 inhibits monocyte differentiation into DC, DC maturation, and DC-mediated T-cell activation [97,98,101]. Tolerogenic DCs have also been shown to express CD83 in order to maintain mucosal homeostasis and the self- versus non-self-immunity control [95,100]. Furthermore, Treg generation seems to rely on sCD83 and indoleamine 2-3 dioxygenase 1 (IDO-1) production by DCs. Lastly, as is the case for NR4As, CD83 is essential for the maintenance of the Treg phenotype [102,103].

### 4.2. Importance of CD39 and CD73

We have previously shown that MZps express high levels of the ectonucleotidases CD39 and CD73, molecules involved in the adenosine (ADO) pathway [4]. As such, CD39 converts the extracellular ATP (highly inflammatory, and notably generated by cell death) into ADP and AMP, and CD73 converts the latter into ADO, an anti-inflammatory molecule [77,104]. Thus, CD39 and CD73 expression allows for the conversion of a pro-inflammatory milieu into an anti-inflammatory one. ADO production has been shown to induce a wide variety of anti-inflammatory responses [105]. For instance, the binding of ADO to the A2_A_ receptor in FO B-cells impedes GC formation, BCR signaling and TLR responses [106]. In T-cells, ADO can promote Treg generation, which will express CD39 and CD73 [107]. Furthermore, A2_A_ signaling (possibly autocrine or paracrine) in Tregs increases IL-10 and TGF-β production by these cells, further nourishing the anti-inflammatory environment generated by the ADO production [108]. As such, CD39 and CD73 expression was evaluated in a wide variety of contexts, including cancer, where these molecules and ADO have been found to contribute to the maintenance of the “cold”, anti-inflammatory tumoral microenvironment [108]. CD39 and CD73 have previously been identified as regulatory molecules on T-cells and B-cells by different groups [105,107]. Indeed, some Breg populations were associated with CD39 and/or CD73 expression [77]. The binding of ADO to the A2_A_ receptor activates adenylate cyclase, allowing for intracellular cyclic AMP (cAMP) production and accumulation, which will then inhibit the NK-κB response and the Janus Kinase (JAK)-Signal Transducer and Activator of Transcription (STAT) pathway, important for inflammatory responses [108]. ADO binding to the A2_A_ receptor has been shown to upregulate NR4A expression in monocytes [84]. Given the importance of cAMP to CREB activation, and thus, to NR4A expression, a link between the adenosine pathway and NR4A expression is to be expected.

## 5. The BAFF/APRIL System

We cannot present MZ B-cell populations without discussing the BAFF/APRIL system. Without a doubt, one of the most important molecules for the survival and differentiation of B-cells is BAFF. BAFF, also known as B lymphocyte stimulator (BLyS), is part of the tumor necrosis factor (TNF) family and is encoded by the *TNFSF13B* gene [109]. BAFF possesses three receptors found across all B-cell populations; they are BAFF-R, TACI and B-cell maturation antigen (BCMA) [110]. The latter two are also shared with the BAFF analog APRIL, encoded by the *TNFSF13* gene, with which it shares a strong homology [110].

BAFF is a transmembrane protein that can be expressed as trimers at the surface of DCs, monocytes, macrophages, activated T-cells and B-cells, neutrophils, and the stroma of secondary lymphoid organs; alternatively, BAFF can be cleaved by a furin protease and released in a soluble form [109]. Interestingly, BAFF in its soluble form can associate with 20 other BAFF trimers and form a 60-mer, a giant virus capsid-like structure that confers different signals when compared to its trimer form [111,112,113]. APRIL can only be found in a soluble form, also in trimers, since its membrane domain is cleaved in the Golgi apparatus as part of its maturation process. Interestingly, APRIL can also complex itself with heparan sulfate proteoglycans (HSPG) such as perlecan, and then bind to its receptors [114]. Furthermore, BAFF and APRIL can form heterotrimers that possess different affinities with receptors of the BAFF/APRIL system [115]. However, the precise involvement of these heterotrimers in immune responsiveness remains to be elucidated.

As previously described, BAFF-R signaling is important for MZ cell fate decision by activating the NF-kB pathway and delivering survival signals [12], and possibly by upregulating NOTCH2 expression [12,21,22]. TACI signaling, on the other hand, is mainly involved in MZ antibody production and CSR (see below) [43]. Lastly, BCMA signals play an important role in plasma cell survival and differentiation [110]. TACI signaling has been shown to reduce the activation threshold of MZ by cross-linking between the TLR pathway and the phosphatidylinositol 3-kinase (PI3K)- protein kinase B (AKT)- mechanistic target of rapamycin (mTOR), PI3K-AKT-mTOR pathway [109,116]. Furthermore, following the binding of BAFF/APRIL to TACI, recruitment of the TNF receptor associated factor (TRAF) ensues, involving TRAF2 and TRAF6, while BAFF-R signaling involves TRAF2 and TRAF3 [109]. Interestingly, TRAF3 has been shown to negatively regulate CREB, possibly modifying the transcriptional program of the B-cell to a more activated state, as the expression of CREB-induced molecules, such as NR4As, are generally related to anti-inflammatory and activation control roles [92,117]. Thus, the BAFF/APRIL system is involved in the shaping of MZ pools and their effector functions. The fact that these factors are often found to be in excess in the context of inflammation is likely to perturb MZ B-cell populations’ homeostasis.

## 6. HIV Infection and the Dysregulation of the B-Cell Compartment

Even if HIV does not infect B-cells directly, the early and persistent inflammation associated with this infection—despite highly active antiretroviral therapy (HAART)—affects virtually all arms of the immune system, including the B-cell compartment [118].

It has been shown that BAFF levels in the blood of HIV infected individuals are in excess when compared to healthy individuals, which correlates with hyperglobulinemia and breakage of tolerance [119]. We and others have shown that excess BAFF persists despite HAART in several different cohorts, as well as in simian immunodeficiency virus (SIV)-infected macaques and HIV-transgenic (Tg) mice [5,120,121,122,123,124]. As such, BAFF is one of several reliable markers of inflammation that correlates with the chronic inflammation associated with HIV infection. There are several reasons that can explain this increase in BAFF levels in HIV-infected individuals, some of which are viral factors and others of which are non-viral factors. First of all, some viral proteins detected despite HAART, such as negative regulating factor (Nef)—an accessory protein that has a key role in HIV infection—or gp120 of the HIV envelope (Env), are capable of directly up-regulating BAFF expression by MoDCs and monocytes, respectively [120,125]. Furthermore, TLR ligands and/or type I interferons (IFNs) such as interferon alpha (IFNα), abundantly produced during viral infections, lead to the production of BAFF [125,126,127]. Excess BAFF can also be caused by non-viral factors such as elements of microbial translocation, e.g., lipopolysaccharides (LPS), shown to promote BAFF expression by MoDCs [120].

Hyperglobulinemia, especially hypergammaglobulinemia (high polyclonal IgG titers in blood) is one of the main characteristics of HIV-associated B-cell deregulation, and is even one of the first ever described in people living with HIV [118,128]. Hyperglobulinemia is caused by the non-specific polyclonal activation of the B-cell compartment as a result of the excessive inflammation associated with the HIV infection context [118]. This state is fueled by the excess of pro-inflammatory cytokines such as IFN-α and TNF-α, which are produced in response to the viral infection itself [120,129]. Microbial translocation associated with massive HIV replication in the GALT also participates in the hyperactivation of the B-cell compartment via PRRs [130,131]. As mentioned above, excessive BAFF signals also favor polyclonal B-cell activation, notably that of innate-like B-cells such as MZ and MZp.

Notably, hyperglobulinemia is also associated with the presence of autoreactive antibodies. Interestingly, excess BAFF has been associated with the production of autoreactive antibodies in autoimmune diseases such as systematic lupus erythematous (SLE), rheumatoid arthritis (RA) and Sjögren syndrome (SS) [132]. This is suggested to be mainly due to the BAFF delivery of survival signals having the capacity to bypass apoptotic signals that would otherwise eliminate autoreactive B-cells during their selection in the periphery [132].

HIV infection is also characterized by the loss of circulating memory B cells, despite HAART [133,134,135]. This phenomenon could be partly explained by the downregulation of expression of BAFF-R by memory B-cells, which is essential for delivering the survival signals needed to keep these cells alive. Furthermore, in the HIV context, memory B-cells also express apoptosis markers such as CD95 (Fas), forkhead box o3 (FOXO3a) and TNF-related apoptosis inducing ligand (TRAIL), which are involved in cell death [136,137,138]. This loss of memory B-cells also affects memory generated in response to childhood vaccination antigens, further nourishing the immune incompetence observed in people living with HIV (PLHIV) [139,140].

Another important factor is the loss of CD4^+^ T-cells, the main targets for HIV. As previously described, memory B cells result from a long process that takes place in the GC, one that requires the implication of CD4^+^ T-cells, notably T_fh_. Without these cells, efficient T-dependent responses cannot take place. In fact, in HIV-Tg mice and BAFF-Tg mice, the formation of GC is impaired and FDC networks reduced, with lowered expression of CD40L by activated CD4^+^ T-cells [141,142,143,144]. Similar observations were seen in the context of human HIV infection [145,146,147,148].

## 7. MZp in the Context of HIV

Our initial work with HIV-Tg mice showed an expanded marginal zone in the spleen of these animals, as well as B-cell hyperactivity and hyperglobulinemia with elevated anti-nuclear auto-antibodies [141]. Interestingly, we found numerous extra-follicular IgM bright plasma-cells in the spleen of these HIV-Tg mice [141]. Notably, BAFF levels were found to be in excess in the serum of these animals [124]. Similar observations have been made with BAFF-Tg mice [144]. In agreement with our findings with HIV-Tg mice, we have shown that frequencies of MZp are increased in the blood of HIV-infected individuals from the Montreal primary HIV infection (PHI) cohort, as soon as in the acute phase, and despite HAART; they are concomitant with excessive BAFF levels which persist throughout, suggesting that deregulations of MZ population frequencies in the HIV context could involve excess BAFF [5,6]. As such, and as mentioned above, BAFF signals are important for the selection of the MZ B-cell pool [13]. The fact that BAFF has been shown to increase the expression of NOTCH2, whose signal is essential to MZ cell-fate decision, suggests that in excessive BAFF contexts, increased NOTCH2 may skew differentiation towards the MZ type, contributing to their increased frequencies [13,23].

Chemokines such as CCL20 and CCL25 were found in excess in the blood of HIV-infected individuals from the Montreal PHI cohort, and MZp from these individuals strongly migrated in response to these chemokines in vitro [149]. CCL20 and CCL25 are important chemokines that allow B-cell migration to peripheral sites such as the mucosal associated lymphoid tissues (MALT) [150,151]. This modulation in MZp migratory capacities could also help explain the increased frequencies of MZp in the blood, as these cells are being actively recruited to peripheral sites; where they are possibly solicited in an attempt to control HIV inflammation in places where the active battle against the virus is held. Notably, populations such as MZ accumulated in lymphoid organs of SIV-infected macaques [152]. Importantly, MZps from the blood of HIV-infected individuals from the Montreal PHI cohort express α4β7, shown to bind to gp120 and be important for mucosal migration (data not published). It is possible that some MZps be naturally recruited to the MALT, where they perform Breg- and antibody-producing activities [152]. As such, the fact that MZ populations are capable of CSR could suggest their being related to the recently reported α4β7 IgA-expressing Bregs, promoted by APRIL via TACI [74,153]. Any disturbance in the activities of such populations is likely to have a deleterious outcome.

Importantly, our recent work shows that the Breg potential of blood MZps from HIV-infected individuals of the Montreal PHI cohort is severely altered despite therapy, and suggests that BAFF may directly contribute to this altered profile. Given the association of excess BAFF with hyperglobulinemia and autoimmune manifestations, it is reasonable to think that in such circumstances, MZps are rather driven to antibody production, the desirability of which is questionable.

**Figure 2:**
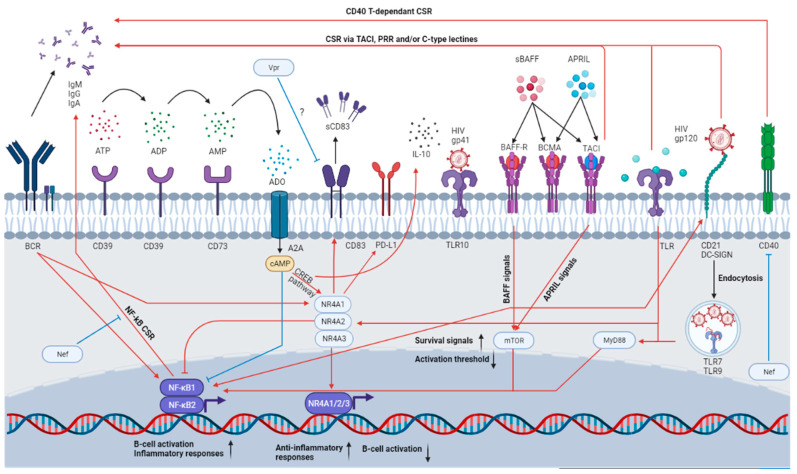
MZp immune functions and how they can be affected in the HIV context. MZps possess a strong Breg potential, as attested by the expression of several immunoregulatory molecules. Indeed, MZps express CD39 and CD73, which will convert the extracellular ATP into ADO. This molecule will then be uptaken by purinergic receptors such as A2_A_, which will induce cAMP accumulation in the cytosol and the activation of the CREB pathway. CREB will induce the expression of CREB-induced elements such as the NR4A molecules and IL-10, which will allow for the maintenance of a regulatory phenotype. The NR4As will then induce the expression of even more immunoregulatory molecules such as CD83 and PD-L1, while also impeding unwanted cell activation by the BCR or the TLR. However, this homeostasis is heavily altered in the HIV context due to the chronic inflammation, excess BAFF and viral proteins. For instance, MZ B-cells are able to class-switch following CD40 engagement and subsequent NF-kB pathway activation. However, in the HIV context, HIV Nef could impede this CSR. HIV gp120 could activate B-cells by cross-linking DC-SIGN an action that is enhanced by BAFF. Excess BAFF could induce TACI-dependent CSR by activating the mTOR pathway, which intersects with the TLR pathway (also engaged due to HIV-mediated recognition by TLR7, expressed by MZ B-cells), lowering the MZ activation threshold. HIV proteins such as Vpr could also directly affect MZp immunoregulatory protein expression such as CD83. Thus, in the HIV context, MZps could possibly lose their immunoregulatory functions, become easily activated and produce poor-affinity antibodies, with possible auto-reactivity.

Interestingly, MZ B-cells were shown to bind to glycoproteins of the HIV Env, such as gp120, via C-type lectins—such as dendritic cell-specific intercellular adhesion molecule-3-grabbing non-integrin (DC-SIGN)—and the mannose receptor, or via their polyreactive BCR; and a fraction of IgG and IgA produced following gp120 stimulation in the presence of BAFF was shown to recognize gp120 [38]. Additionally, the stimulating effect of gp120 on MZ populations is enhanced in the presence of BAFF [38]. MZ B-cell populations can also recognise HIV Env proteins such as gp41 through TLR10 and CD21 via the complement system [154,155] (see Figure 2). However, the exact contribution of MZ and MZp to anti-Env Abs and/or to hyperglobulinemia and auto-antibodies needs further assessment.

HIV proteins can directly affect MZ and MZp capacity and function. For instance, it has been shown than soluble Nef, possibly produced and released by the HIV reservoirs, penetrates B-cells and directly impedes CD40 signaling mediated through the NF-kB and STAT pathway, and thus, CSR [156]. Moreover, the HIV Viral protein R (Vpr) has been shown to downregulate CD83 expression in both macrophages and DCs [157,158]. Furthermore, as described above, HIV Env glycoproteins can directly activate MZ populations (Figure 2).

Lastly, consistent with the notion that they are highly solicited, MZps from the blood of HIV-infected individuals present an exhausted profile; this is depicted by the upregulation of the negative regulators CD22 and CD72, as well as the exhaustion markers CD85j and FCRL5. The expression of T-bet and CD11c were also upregulated by these MZps. Interestingly, T-bet and CD11c expression are related to extra-follicular B-cell responses and to a population identified as “age-associated B-cells”; these are also described in the contexts of chronic inflammation and autoimmunity (discussed below), and are dependent on IL-21R and TLR7 signalling [159]. Age-associated B-cells are thought to produce antibodies of poor affinity. Interestingly, MZps express both IL-21R and TLR7, which means that they have the potential to take part in the age-associated B-cell pool.

## 8. MZps and Similar Populations in Other Diseases

### 8.1. Autoimmune Diseases

As mentioned above, in the HIV context, MZps share some similarities with a heterogeneous T-bet^+^CD11c^+^ population reported to be increased in the context of chronic infections and inflammatory conditions; which profile is reminiscent of “age-associated B-cells”, a cell population first described in mice [160,161]. These T-bet^+^CD11c^+^ cells have been associated with disease progression and clinical manifestations in SLE patients [162,163]. Interestingly, women are more affected than men by autoimmune diseases [164]. It is known that estrogen promotes the activation and expansion of autoreactive MZ B-cells in both mice and humans [2].

As we have observed for the Breg profile of blood MZp from HIV-infected individuals, deregulations of different Breg populations are also associated with autoimmunity, such as those observed in SLE, multiple sclerosis (MS) and RA [165]. For example, CD19^+^CD24^high^CD38^high^ B-cells from the blood of SLE-afflicted individuals lose the capacity to control TNF-α and IFN-γ production by CD4^+^ T cells [68]. Similarly to the HIV context, in all these cases, BAFF was found in excess, and played a role in the development of autoimmunity [68,132,165,166]. In fact, in SLE and SS, excess BAFF positively correlates with the level of circulating auto-antibodies [132].

### 8.2. Atherosclerosis

Chronic inflammation in PLHIV has been associated with the premature development of age-associated comorbidities such as atherosclerosis, the main risk factor for cardio-vascular disease (CVD) [167,168,169]

Atherosclerosis is, by its nature, an inflammatory disease; and the persistent chronic inflammation that prevails in PLHIV may fuel its early development. As such, when matched for traditional risk factors, HIV-infected individuals had a higher chance of developing CVD when compared to HIV-uninfected individuals [167,168,170].

The role of BAFF in atherosclerosis development is complicated and poorly explored in humans (most of the research was traditionally conducted in mice). For instance, in the aforementioned research, BAFF neutralization aggravates atherosclerosis, while BAFF overexpression attenuates this disease [171,172]. This has been attributed to TACI-expressing cells, such as MZ B-cells, which express high levels of this receptor. Indeed, MZ B-cells were shown to possess an atheroprotective role due to PD1-PD-L1 interactions with T_fh_ cells, allowing for a better control of GC reactions, a role that was attributed to MZ B-cell NR4A1 expression [173,174]. Moreover, FO B-cells are considered to be atherogenic, as they generate GC responses and, subsequently, IgG directed against oxidized LDL (oxLDL) [175]. Thus, in the context of MZ and MZp deregulation, such as the one found in HIV infection, it is possible to assume that these cells lose their capacity to maintain their immune surveillance capacities, contributing to the early onset of atherosclerosis in HIV-infected individuals.

In humans, excess BAFF was also found to correlate with CVD development in autoimmune diseases such as SS and SLE. As a matter of fact, CVD was found to be the major cause of death in individuals afflicted by SLE [176,177]. As such, BAFF, when in excess, could be related to the premature development of CVD; this can either be directly—through it being overtly produced by adipocytes and acting as an adipokine linking obesity and inflammation, and by contributing to the apoptosis of endothelial cell progenitors (a process known as endothelial dysfunction, a triggering factor for atherosclerosis development)—or indirectly, by altering the atherosclerosis immune surveillance processes, which are usually warranted by Breg populations such as MZ B-cell populations [178,179].

### 8.3. Severe Acute Respiratory Syndrome Coronavirus 2 (SARS-CoV2) and Other Viral Infections

It was found that individuals who had died of the SARS-CoV-2 infection had a lack of GC in their lymphoid organs, which was partly explained by the downregulation of the transcription factor B-cell lymphoma 6 (Bcl-6) by B-cells and T-cells [180]. This loss of GC was associated with increased frequencies of T-bet^+^CD11c^+^ extra-follicular B-cells, which have been associated with a strong production of auto-antibodies and poor disease outcome in individuals infected with SARS-CoV-2 [181]. Indeed, auto-antibodies directed against interferons are one of the key triggering events to critical COVID-19 pneumonia and death in patients who develop this disease [182]. Unsurprisingly, BAFF was found to be elevated and to persist in individuals with severe disease [183]. Of interest is the fact that levels of APRIL were found to be elevated in individuals who had recovered from the infection [183,184].

Reports of an extra-follicular population sharing similar features with MZp, known as CD21^low^ MZ-like B-cells, was found to be increased in individuals infected with hepatitis C virus (HCV) [185]. This population expressed an autoreactive BCR and was correlated with increased autoimmunity in the HCV context [185].

Overall, most chronic inflammatory conditions are associated with excessive BAFF levels and polyclonal B-cell activation, at the expense of functional immune surveillance. If not addressed therapeutically, these could lead to long term and/or persistent autoimmune manifestations and life-threatening co-morbidities.

### 8.4. Malignancies Associated with MZ Deregulations

One complication often associated with deregulations of MZ B-cell populations is Marginal Zone Lymphoma (MZL), which is the second most common non-Hodgkin’s lymphoma and which possess varying manifestations (according to the WHO classification), such as splenic MZL, nodal MZL and extra-nodal MZL of the MALT, depending on the initiating site [186,187]. Many of these lymphomas appear due to mutations on genes associated with MZ differentiation, such as *NOTCH2*, as well as mutations on genes involved in the BCR signaling and NF-kB pathways [188,189]. The differential diagnosis between the myriad of different MZL manifestations is complex and requires several investigations, notably immune profiling and genetic tests [190].

Non-Hodgkin’s lymphomas are highly represented in PLHIV. Even though MZL is not an AIDS-defining lymphoma, its incidence is indeed higher when compared to healthier populations [191,192]. In PLHIV, the immune-incompetence caused by the HIV-infection and chronic inflammatory condition, despite HAART, may be involved in the development of these types of lymphomas. As such, chronic inflammation and autoimmune manifestations were found to be related to the development of MZL malignancy in PLHIV as well as in individuals diagnosed with SLE, SS and RA [193,194]. Additionally, MZL development has been associated with chronic infection by *Helicobacter pylori* and *Borrelia burgdoferi* in the case of gastric MZL and subcutaneous MZL, for instance [191,195]. Interestingly, certain types of MZL are also associated with a “biased” usage of Ig heavy chains, implying that the capacity to respond to certain types of antigens is a predicting risk for the development of these diseases [196,197]. Moreover, it has been shown that CSR and SHM, mediated by the upregulation of Activation-induced cytidine deaminase (AID) due to inflammation and increased NF-κB expression, induce genomic instability, driving carcinogenesis [198]. Thus, chronic activation of MZ B-cells in the context of autoimmunity or in the HIV context, for instance, could be a triggering factor for the development of this type of malignancy. As such, our recent report that NR4As are severely and significantly downregulated in blood MZp from HIV-infected progressors may constitute prognostic markers for MZL development in these individuals, as NR4A1 has been reported to be severely downregulated in aggressive and indolent human B-cell lymphomas [199].

One of the key phenotypical differences between FO and MZ B-cells is the expression of IgD, the latter expressing lower levels of this molecule than the former [1]. However, in certain types of MZL, such as splenic MZL, tumor cells heavily express IgD, which could be used as a marker to distinguish splenic MZL cells from other types of MZL that happened to invade the spleen [200]. Notably, it has been shown that in a model of constitutive induction of NOTCH2, FO B-cells could differentiate into MZ B-cells [19]. As such, mutations triggering the expression of this molecule or mutations in its master regulator, Kruppel-like factor 2 (KLF2)—both of which were found in MZL—could be related to the generation of atypical MZ, leading to the development of this type of cancer [191,201]. Interestingly, the constitutive induction model of NOTCH2 induced a strong downregulation of KLF2 [19]. Thus, more studies need to be conducted in this field.

## 9. Possible Therapeutic Avenues

Since HAART is not sufficient to cease chronic inflammation and the associated development of co-morbidities and autoimmune manifestations in HIV-infected individuals, the addition of other drugs could be contemplated as an adjunct to HAART; this might help to lower the inflammatory burden and restore immune competence, especially given the fact that, nowadays, those individuals live longer. In this view, lowering BAFF levels with reagents such as the FDA-approved Belimumab (Benlysta) could be contemplated, as this antibody is currently used in the treatment of SLE and shows satisfactory results in the improvement of disease progression [202]. Other drugs, such as dihydroergotamine (DHE), that upregulate NR4A expression levels, have potential in treating acute myeloid lymphoma (AML) through induction of apoptosis of cancerous cells; they could be tested to try to either restore the Breg function of MZps or to induce their apoptosis [203].

## 10. Conclusions

In conclusion, MZps are an important Breg subset that participates in immune surveillance and defense of the organism. However, the equilibrium between these functions can easily be disrupted in chronic inflammatory diseases, as an excess of pro inflammatory molecules such as BAFF can affect both their Breg function and immune surveillance capacities, as well as the nature of the antibodies they produce. A better understanding of the mechanisms regulating MZp functions could benefit to innovative therapeutic strategies viewed to harness this precious cellular potential, to prevent its deregulation, or restore its immune competence.

## Figures and Tables

**Table 1:** Characteristics of several Breg populations in mice and humans.

Species	Population	Phenotype	Mechanism of Suppression	References
Mouse	B10	CD19 + CD5 + CD1d^hi^	IL-10	[52,53]
	MZ B-cells	IgM^hi^ IgD^lo^ CD21^hi^ CD23-CD1d^hi^	IL-10	[54]
	T2-MZP	B220 + CD21^hi^ CD1d^hi^ IgM^hi^ CD23+	IL-10	[55]
	B1a	CD90-CD5+	IL-10	[56]
	Plasma cells	CD19 + CD138 + IgM+	IL10, IL-35	[57]
	Plasmablasts	CD138 + CD44^hi^	IL-10	[58]
	Tim-1 + B-cells	CD19 + Tim-1+	IL-10	[59]
	IL-35-Bregs	CD5 + CD1d^hi^ FcγIib^hi^	IL-35	[60]
	GITRL + B-cells	-	GITRL	[61]
	Killer B-cells	CD19 + CD5 + FasL+	FasL, TGF-β	[62,63]
	PD-L1^hi^ B-cells	CD19 + PD-L1^hi^	PD-L1	[64]
	-	B220 + CD39 + CD73+	ADO, CD39 + CD73 + Extracellular vesicules	[65,66]
	GIFT-15 B-cells	B220 + CD21 + CD22 + CD23 + CD24 + CD1d + CD138 + IgM + IgD+	IL-10	[67]
Human	MZp	CD19 + CD1c + CD21^lo^ IgM^hi^ CD27 + CD10+	CD83, PD-L1, IL-10	[4,6]
	Transitional B-cells	CD19 + CD24^hi^ CD38^hi^	IL-10	[68]
	Memory B-cells	CD19 + CD24^hi^ CD27+	IL-10	[69]
	Br1	CD25^hi^ CD71hi CD73^lo^	IL-10	[70]
	TIM1 + B-cells	CD19 + TIM1+	IL-10	[71]
	Plasmablast	CD19^lo^ CD27^hi^ CD38^hi^	IL-10	[72,73]
	IgA + B-cells	CD19 + IgA+	IL-10, PD-L1	[74]
	Exhausted B-cells	CD19 + CD95+	CD95	[75]
	Killer B-cells	CD19 + CD38 + IgM + FasL+	FasL	[76]
	PD-L1 B-cells	CD19 + PD-L1+	PD-L1	[63]
	CD39^high^	CD19 + CD39^high^CD73+	ADO	[77]
	iBreg	-	TGF-β, IDO	[78]

The phenotype and mechanism of suppression of different Breg subsets in mice and humans are summarized herein.

**Table 2:** The regulatory molecules expressed by human blood and tonsillar MZps.

mRNA Expression	Confirmed Protein Expression
NR4A1, NR4A2, NR4A3, CD83 CD39, CD73, TGF-β, IL-10, PD-L1, IL-10R, IL-27β, IL-12 p35, HLA-G	NR4A1, NR4A3, CD83, CD39, CD73, PD-L1, IL-10

Human blood MZps express high levels of mRNA transcripts of genes associated with a regulatory potential. A certain number of these regulatory transcripts had their protein expression confirmed, both in blood and tonsillar MZps [4,6].

## Data Availability

Not applicable.
